# A Lab-on-a-Chip-Based Non-Invasive Optical Sensor for Measuring Glucose in Saliva

**DOI:** 10.3390/s17112607

**Published:** 2017-11-13

**Authors:** Dong Geon Jung, Daewoong Jung, Seong Ho Kong

**Affiliations:** 1 Graduate School of Electronics Engineering, Kyungpook National University, Daegu 41566, Korea; max700128@hanmail.net; 2 Aircraft System Technology Group, Korea Institute of Industrial Technology (KITECH), Daegu 42994, Korea; dwjung@kitech.re.kr

**Keywords:** glucose, optical sensor, saliva

## Abstract

A lab-on-a-chip (LOC)-based non-invasive optical sensor for measuring glucose in saliva was fabricated. Existing glucose sensors utilizing blood require acquisition of a blood sample by pricking the finger, which is painful and inconvenient. To overcome these limitations, we propose a non-invasive glucose sensor with LOC, micro-electro-mechanical system and optical measurement technology. The proposed sensor for measuring glucose in saliva involves pretreatment, mixing, and measurement on a single tiny chip. Saliva containing glucose and glucose oxidase for glucose oxidation are injected through Inlets 1 and 2, respectively. Next, H_2_O_2_ is produced by the reaction between glucose and glucose oxidase in the pretreatment part. The saliva and generated H_2_O_2_ are mixed with a colorizing agent injected through Inlet 3 during the mixing part and the absorbance of the colorized mixture is measured in the measurement part. The absorbance of light increases as a function of glucose concentration at a wavelength of 630 nm. To measure the absorbance of the colorized saliva, a light-emitting diode with a wavelength of 630 nm and a photodiode were used during the measurement part. As a result, the measured output current of the photodiode decreased as glucose concentration in the saliva increased.

## 1. Introduction

The incidence of diabetes is increasing, and over 400 million people are currently affected worldwide. The number of people with diabetes is expected to increase to 600 million by 2040 [1]. Patients with diabetes have a greater risk of critical health problems compared to non-diabetic individuals. Critical diseases involving the heart or blood vessels are induced by high blood glucose concentrations. Therefore, accurate monitoring of blood glucose concentration in patients with diabetes is important for real-time diagnosis and management of diabetes [2,3,4,5]. Existing conventional methods for monitoring blood glucose concentration requires acquiring a blood sample by pricking the finger. However, this method is painful, and several blood samples are needed several times each day. Thus, many researchers have investigated the development of non-invasive glucose monitoring systems utilizing body fluids such as saliva, tears, and sweat without of the need for a blood sample [6,7,8,9]. Saliva has various advantages compared with other body fluids. First, it is easily tested by individuals without special training; second, the risk of infection or cross-contamination is low because frequent finger pricking is not required; third, it is convenient for patients with diabetes who face difficulty extracting blood samples such as infants, the elderly, and hemophiliacs; finally, previous studies have demonstrated a good correlation between blood glucose concentration and saliva glucose concentration. Therefore, continuous monitoring of glucose concentration in the blood may be achieved by tracking the saliva glucose concentration. The point to be careful of here is that a non-invasive glucose sensor utilizing saliva should detect milligram concentrations of glucose, as the glucose concentration in the saliva of diabetic patients is several hundred-fold lower than that in the blood [10,11]. However, reported technologies for noninvasively detecting glucose are expensive and complicated to fabricate [11]. In general, estimating technologies with saliva consist in optical measurement methods such as liquid chromatography-mass spectrometry (LC-MS) and UV-Vis spectrophotometry. However, these optical measurement systems can only be used in a laboratory because they require significant processing time, expensive reagents, sophisticated instruments, and highly trained professionals. Thus, it is difficult to use individual glucose monitoring at home or in daily activities [12,13,14]. Therefore, a novel glucose sensor that is sensitive, accurate, convenient, low-cost, and non-invasive is needed. One way to satisfy these demands is to use lab-on-a-chip (LOC) technology. An LOC technology enables several laboratory functions such as mixing, pretreatment, and measurement on a single chip. LOC technology has shown remarkable development for many applications in various areas, such as chemical analysis, environmental monitoring, and medical diagnosis [15,16,17,18,19]. This technology has many advantages, such as very small amounts of sample and reagent consumption, rapid detection, and biological and chemical compatibility. Thus, LOC technology was applied to develop non-invasive glucose sensor utilizing saliva in this study. 

The proposed LOC-based non-invasive optical sensor for measuring glucose in saliva consists of a mixing part to mix saliva with reagents, a pretreatment part to separate glucose from saliva, and a measurement part to analyze glucose concentration in the saliva utilizing an optical measurement method. We investigated optimal and novel methods for improving the performance of the proposed LOC-based non-invasive optical sensor for measuring glucose in saliva. First, a mixing part was designed to enhance mixing efficiency by modifying the micro-channel structure. Second, a colorimetric method was utilized to optically analyze glucose in saliva. Third, a method for estimating the colorized sample’s absorbance was proposed by utilizing a light-emitting diode (LED) and a photodiode, a novel structure with a 45° reflective surface that increased the optical path length. The proposed glucose sensor was fabricated using a micro-electro-mechanical system (MEMS) and LOC technology and characterized by measuring absorbance of colorized saliva containing glucose. We confirmed that the proposed LOC-based non-invasive optical sensor for measuring glucose in saliva is suitable as a portable device for continuous glucose monitoring.

## 2. Principle of Glucose Detection

**Figure 1:**
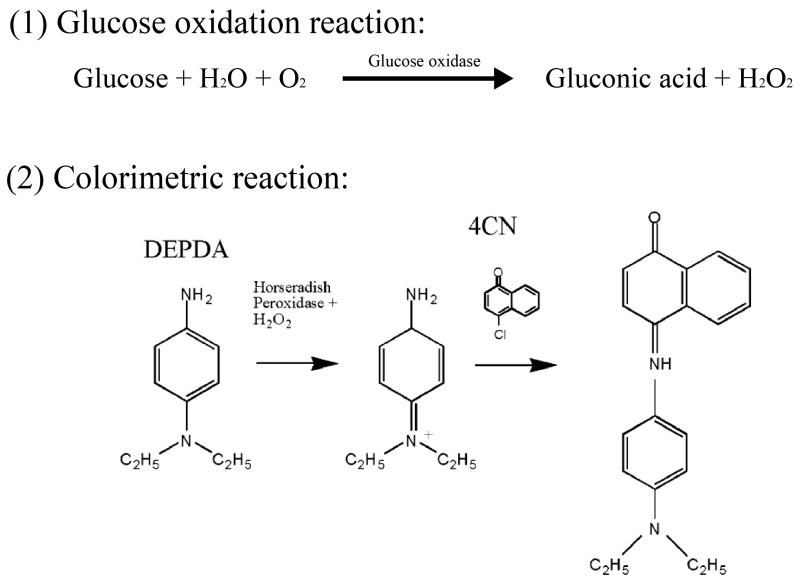
Glucose oxidation and colorimetric reaction utilized in this paper.

Glucose is the most important energy source in the human body and is typically present at a concentration of approximately 0.1%. However, glucose concentration in diabetes patients is significantly increased, which may affect the heart or blood vessels. Thus, detecting glucose without complex and inconvenient procedures is very important. To meet the conditions described above, we investigated a novel LOC-based non-invasive optical sensor for measuring glucose in saliva, a glucose oxidation reaction, an enzymatic colorimetric method, and an optical measurement method. Figure 1 shows the principle of the glucose oxidation process and the colorimetric method [20]. Glucose oxidase converts glucose in saliva to gluconic acid and hydrogen peroxide (H_2_O_2_). Thus, the concentration of glucose is proportional to that of H_2_O_2_ produced by glucose oxidation. Next, a mixture of *N*,*N*′-diethyl-*p*-phenylenediamine (DEPDA), 4-chloro-1-naphthol (4CN), and horseradish peroxidase (HRP) is used to colorize the saliva in accordance with the concentration of the produced H_2_O_2_. HRP is an enzyme catalyst in the glucose oxidation reaction and catalyzes the decomposition of H_2_O_2_ into water. As a result, the saliva turns blue and the absorbance of the blue-colorized saliva is optically measured by the proposed LOC for non-invasive glucose analysis.

## 3. Design and Fabrication

The proposed sensor for monitoring glucose in saliva with LOC, MEMS technology, and optical measurement method was integrated and downsized to include various laboratory functions such as pretreatment, mixing, and measurement processes. Many LOC-based sensors are used in a wide range of biomedical and other analytic applications, including rapid pathogenic organism detection, clinical diagnostics, forensic science, flow cytometry, blood biochemistry tests, and protein and DNA tests. LOC-based sensors can be constructed using MEMS technology and various materials, such as silicon, glass, various polymers, and mixtures of these materials [17].

The proposed LOC-based non-invasive optical sensor for measuring glucose in saliva was divided into three main parts: (1) a pretreatment part, (2) a mixing part, and (3) an absorbance measurement part. The purpose of the pretreatment part is to produce H_2_O_2_, which is proportional to glucose concentration. H_2_O_2_ is produced by a chemical reaction between glucose and glucose oxidase. The produced H_2_O_2_ is mixed with a colorizing agent, which is a mixture of DEPDA and 4CN in the mixing part. The colorizing agent turns blue in the presence of H_2_O_2_ and the absorbance of the colorized mixture is optically measured in the measurement part. 

**Figure 2:**
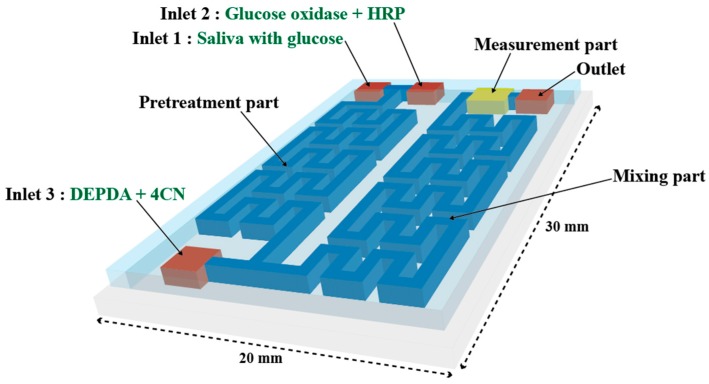
Schematic of the proposed lab-on-a-chip (LOC)-based non-invasive optical sensor for measuring glucose in saliva.

**Figure 3:**
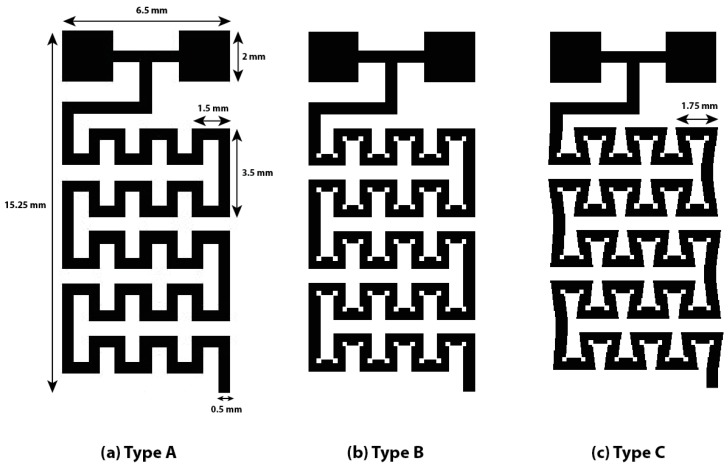
The proposed micro-channel with different structures in order to improve mixing efficiency. (**a**) Type A without obstacles, (**b**) Type B with obstacles, (**c**) Type C with obstacles and the more curvature.

**Figure 4:**
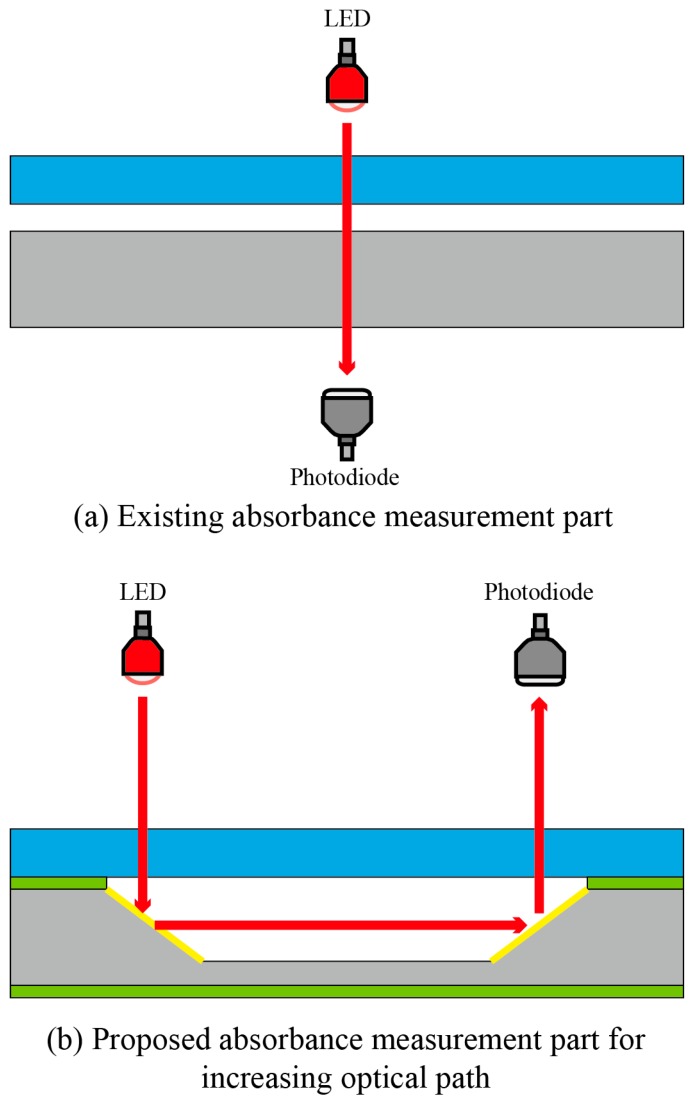
(**a**) The existing absorbance measurement part with (**b**) the proposed absorbance measurement part.

Figure 2 shows a schematic of the proposed LOC-based non-invasive optical sensor for measuring glucose in saliva. The size of the proposed LOC device is 30 mm (L) × 20 mm (W) × 5 mm (H), the channel height is 200 µm, and the channel width is 500 µm. The saliva to be analyzed and a mixture of glucose oxidase and HRP are injected through Inlets 1 and 2, respectively, and injected saliva produces H_2_O_2_ in proportion to glucose concentration in the pretreatment part. Next, the colorizing agent containing DEPDA and 4CN is injected into Inlet 3. The micro-channel in the pretreatment and mixing parts is designed to increase mixing efficiency by installing obstacles inside microchannel. In general, fluid is easily mixed at the macro-scale because it is dominated by turbulent flow, while the mixing of fluids at the micro-scale is not efficient because of laminar flow. Two common methods are used to induce turbulent flow at the micro-scale: for the passive mixing method, obstacles are installed in the micro-channel; in the active mixing method, the sample is physically stirred. To utilize the active mixing method in the proposed sensor, fabrication was quite difficult because micro-actuator must be installed in a micro-channel, additionally. Therefore, in this study, obstacles were installed in the pretreatment and mixing parts to generate a turbulent flow. Figure 3 shows the proposed micro-channel with various structures in order to improve efficiency. Generally, when the direction of the progress of the injected fluid is changed along with the curvature of a two-dimensional structure, the mixing efficiency could be increased because of the centrifugal force at the curvature in the micro-channel. Obstacles are also installed at the curvature in the proposed micro-channel. Saliva is mixed with the injected colorizing agent in the mixing part and flowed into the absorbance measurement part. An LED and a photodiode are installed in the absorbance measurement part to estimate the absorbance of the colorized saliva. In the absorbance measurement part, a 45° angle-etched silicon substrate was designed to improve sensitivity by increasing the optical path length as shown in Figure 4. As mentioned above, the proposed glucose sensor detects glucose concentration in saliva by measuring the intensity of the transmitted light. The emitted light passes the colorized sample and its intensity is reduced along with the concentration of glucose and the optical path length. Thus, increasing optical path length is the best way to improve the sensitivity of the proposed sensor. An increased optical path length was achieved by reflecting incident light several times through the 45° etched reflective surface on the Si substrate. The pretreatment and mixing part designed for analyzing optimally and the absorbance measurement part with the increased optical path were integrated into a tiny single chip.

**Figure 5:**
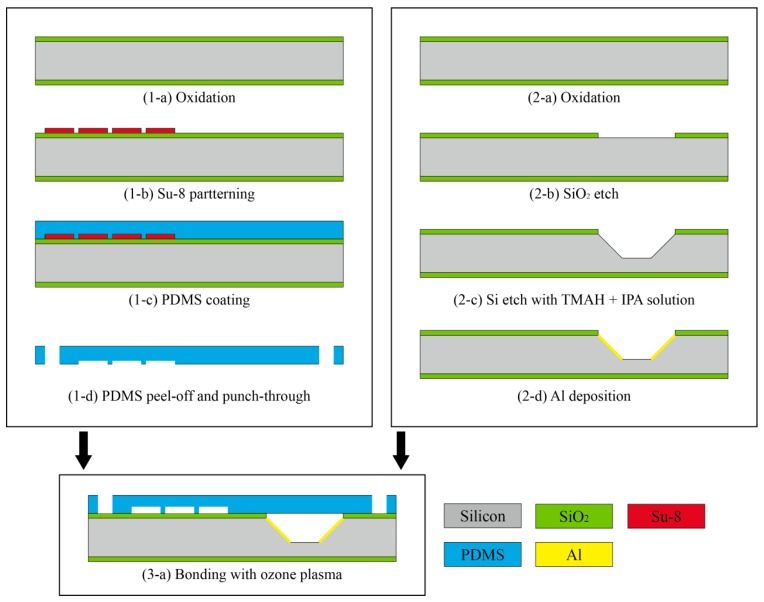
The fabrication process of the proposed LOC-based non-invasive optical sensor for measuring glucose in saliva.

**Figure 6:**
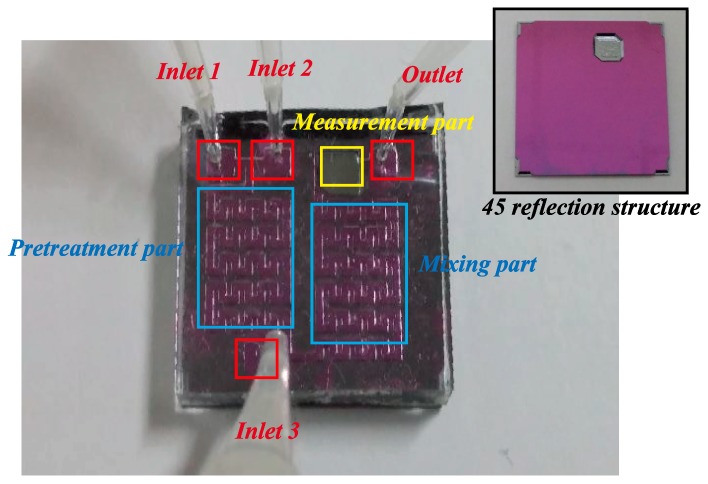
Image of fabricated LOC-based non-invasive optical sensor for measuring glucose in saliva.

Figure 5 shows the fabrication process of the proposed LOC-based non-invasive optical sensor for measuring glucose in saliva. The entire fabrication process consists of three steps: (1) a pretreatment and mixing part fabrication step; (2) an absorbance measurement fabrication step; and (3) a bonding step. First, the SU-8 mold was patterned on the silicon substrate to fabricate the pretreatment and mixing part utilizing a photolithography process. Polydimethylsiloxane (PDMS) was poured onto the silicon substrate-patterned SU-8 mold and cured. PDMS is frequently used to fabricate microfluidic devices because it is transparent, bio-compatible, inexpensive, and easy to fabricate. Next, holes for the inlet and outlet were formed by a punch-through process. Second, the silicon substrate was anisotropically etched by utilizing a mixture of tetramethylammonium and isopropyl alcohol to form a 45° reflective surface to fabricate the absorbance measurement part. Finally, the fabricated pretreatment and mixing parts were bonded to the fabricated absorbance measurement substrate by utilizing ozone plasma for 30 min. Figure 6 shows an image of the fabricated LOC-based non-invasive optical sensor for measuring glucose in saliva.

## 4. Results and Discussion

**Figure 7:**
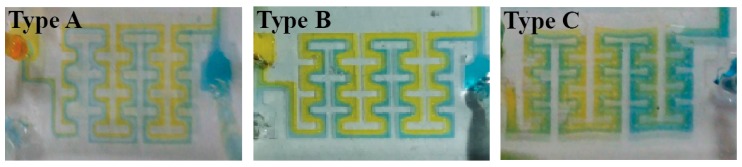
Differences of mixing efficiency along with various micro-channel structures.

First, the performance of the proposed micro-channel with a different structure was estimated. Bromothymol blue (BTB) and pH 8 buffer solutions were used to estimate mixing efficiency of the proposed micro-channel. When BTB and the pH 8 buffer solution were mixed, the mixture turned blue. By utilizing Micropump (model: BT100-1F, Longer pump), BTB and pH 8 solutions were injected into the fabricated micro-channels, here named Types A, B, and C. Figure 7 shows the differences in mixing efficiency along with various micro-channel structures. The micro-channel of Type C, which is the most curved and has the most obstacles, shows the best mixing performance because it induced the most centrifugal force.

**Figure 8:**
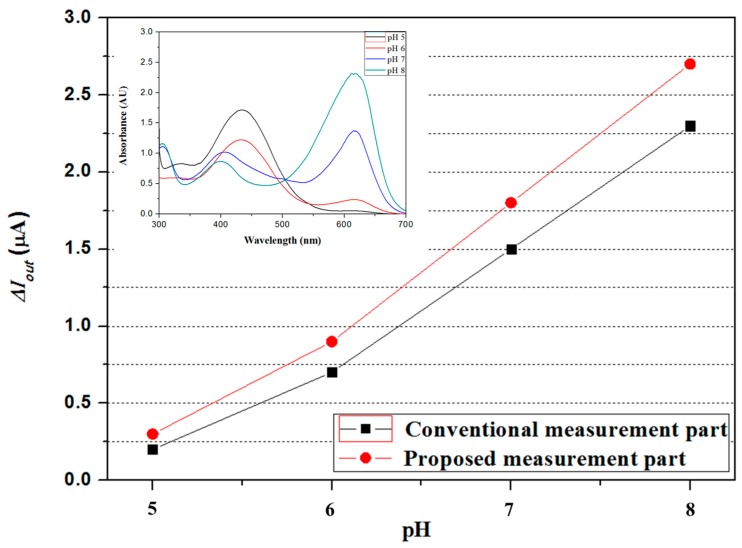
Comparison of the conventional measurement part and the proposed measurement part.

Second, the performance of the proposed optical measurement part with a 45 degree reflective structure was also estimated and compared to that of the conventional measurement part. To estimate the performance of the proposed measurement part, the BTB and pH buffer solutions were firstly mixed, and the mixture’s absorbance was measured utilizing a UV-Vis spectrometer (model: UV-1601, Shimadzu, Inc., Kyoto, Japan) The LED and the photodiode, which matched the measured maximum absorption wavelength range, were used, and the difference between the output currents of the proposed and conventional measurement parts was measured. The output current of the proposed measurement part with a 45 degree reflective structure was greater, as shown in Figure 8.

**Figure 9:**
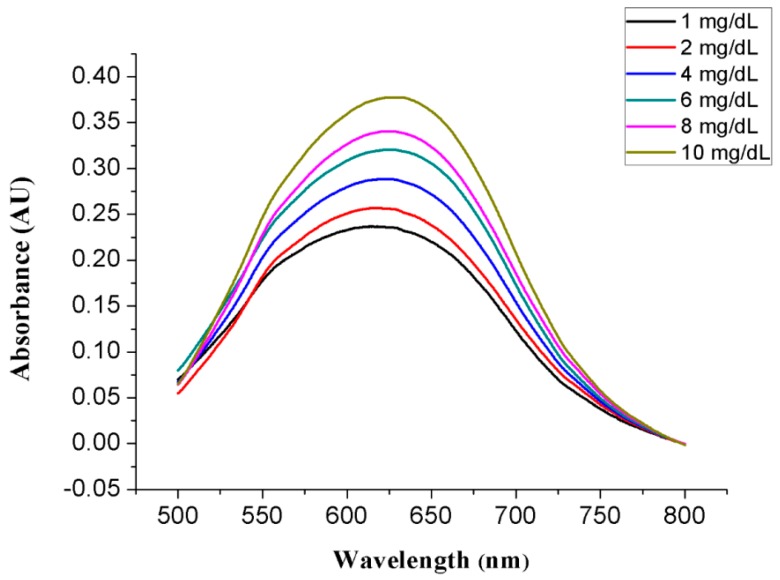
Measured absorbance of colorized sample with different concentrations using a UV-Vis spectrometer.

**Figure 10:**
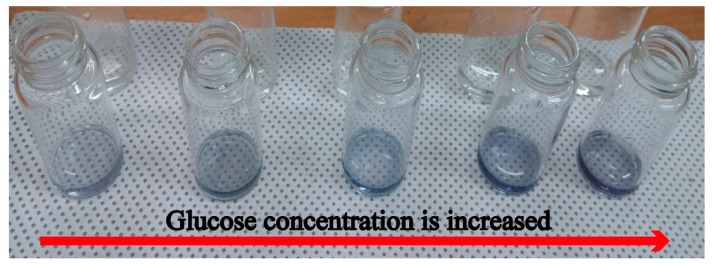
Change of color density as a function of glucose concentration.

Finally, the proposed non-invasive optical sensor for measuring glucose was fabricated and characterized. Before the absorbance of the colorized saliva containing the produced H_2_O_2_ was measured by utilizing the absorbance measurement part on the proposed sensor, a colorized standard glucose solution was measured with a UV-Vis spectrometer (model: UV-1601, Shimadzu, Inc., Kyoto, Japan). A standard glucose solution of a known concentration and a pretreatment agent containing 100 mg/mL glucose oxidase and 20 µg/mL HRP at a 1:1 ratio was injected through Inlet 1, and a colorizing agent containing 1 M 4CN and 1 M DEPDA at a 2:1 ratio was injected through Inlet 3. Next, the absorbance of the colorized standard glucose solution at various concentrations was measured with a UV-Vis spectrometer. Figure 9 shows the measured colorized standard glucose solution absorbance. The measured maximum absorbance was achieved at a wavelength of 630 nm and increased as a function of glucose concentration because more H_2_O_2_ is produced at higher glucose concentrations, leading to a higher color density of the colorizing solution, as shown in Figure 10. A red LED with a wavelength of 630 nm and a photodiode with a measurable wavelength range of 450–1050 nm were used on the proposed measurement part for absorbance analysis. LED emits light at a wavelength of 630 nm and the emitted light passed through colorized saliva. Next, the light reached the photodiode on the proposed LOC, which converted light into current. Therefore, the absorbance as a function of glucose concentration could be estimated from the measured output current and the Beer–Lambert law. 

**Figure 11:**
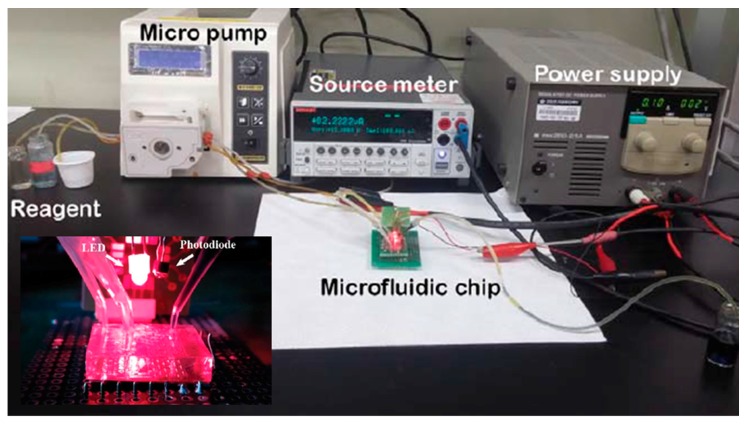
Instrument set-up for the proposed glucose sensor.

**Figure 12:**
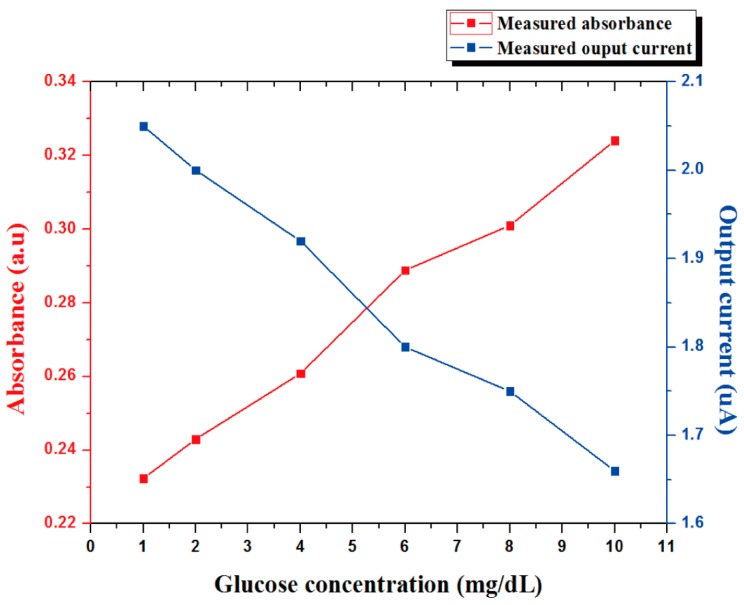
Measured absorbance as a function of the concentration of glucose at a wavelength of 630 nm.

Figure 11 shows the instrument set-up for the proposed glucose sensor. The micropump (model:BT:100 = 1F, Longer pump) is used to flow reagent, and the source meter (SourceMeter 4ZA4, Keithley, Inc., Cleveland, OH, USA) is used to measured the output current along with the glucose concentration in saliva. Figure 12 shows the output current of the photodiode as a function of glucose concentration at a wavelength of 630 nm, and absorbance is derived via the Beer–Lambert law. The output current of the photodiode was decreased as glucose concentration increased in the saliva because of the reduced light transmission.

**Figure 13:**
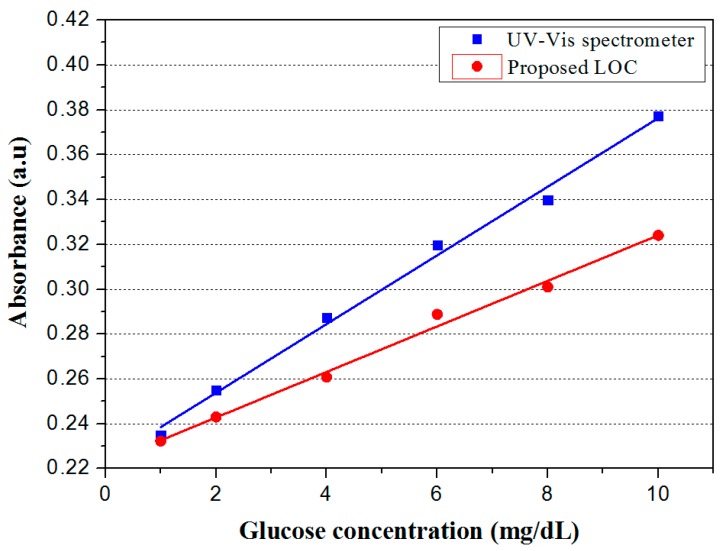
Measured absorbance of proposed LOC and UV-Vis spectrometer as a function of glucose concentration.

Figure 13 shows the measured absorbance along with the glucose concentration by the proposed LOC-based non-invasive optical sensor for measuring glucose in saliva and the UV-Vis spectrometer. Despite the small error in measured absorbance between the proposed LOC and the UV-Vis spectrometer, we confirmed that the analytical performance of the proposed glucose sensor was similar to that of the UV-Vis spectrometer. Thus, the experimental data measured by the proposed LOC-based optical sensor for measuring glucose in saliva was reliable. However, the error between the proposed LOC-based optical sensor for measuring glucose in saliva and the spectrometer was observed due to experimental variables such as the temperature and humidity of the laboratory, loss of light, etc. Particularly, loss of light was induced when light emitted from the LED passed through the PDMS, and the intensity of the light reaching the photodiode decreased as a result. 

**Figure 14:**
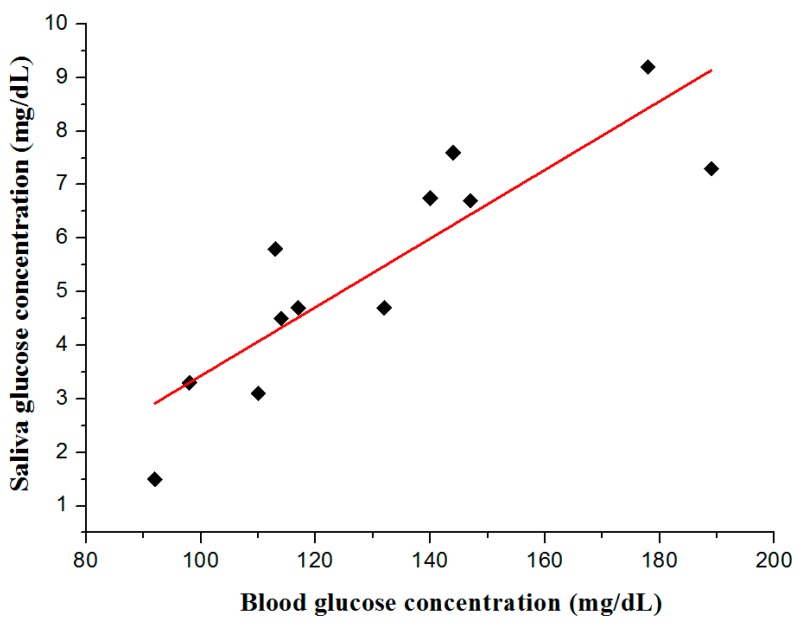
Relationship between blood sugar level and glucose concentration of human saliva using the proposed LOC-based optical sensor.

Finally, we measured glucose concentrations in the blood using a commercial glucose sensor and compared them to measurements using the proposed LOC to estimate the correlation between glucose concentration in the blood and that in the saliva. To estimate the correlation between glocuse concentration in the blood and that in saliva, blood and saliva was collected at the same time, and their glucose concentration was measured with the proposed sensor and the commercial sensor. Figure 14 shows the relationship between glucose concentration in the blood using a commercial sensor (Mirae glucose sensor 3.3G+, measurement range: 10~600 mg/dL, Infopia Co. Ltd., Anyang, Republic of Korea) and that in the saliva using the proposed LOC. Glucose concentration in the saliva increased as a function of glucose concentration in the blood. The experimental results confirmed the relationship between glucose concentrations in the blood and in the saliva and suggests that the proposed glucose sensor can replace the existing sensor. However, additional experiments should be conducted to further optimize the performance of the proposed LOC-based non-invasive optical sensor for measuring glucose in saliva.

## 5. Conclusions

In this study, a single tiny sensor for non-invasive glucose analysis was proposed and fabricated by utilizing LOC, MEMS technologies, and an optical measurement method to replace existing invasive glucose analysis devices. The proposed LOC-based optical sensor for measuring glucose in saliva consists of a pretreatment part for the glucose oxidation reaction, a mixing part for colorizing the pretreated saliva, and a measurement part for measuring absorbance of colorized saliva using an LED and a photodiode. The proposed sensor utilizing saliva is non-invasive, small, cheap, and durable and can be used for real-time analysis. The most important consideration when using saliva as an experimental sample is sensitivity, as the glucose concentration in the saliva of diabetic patients is several hundred-fold lower than that in the blood. Thus, the pretreatment and mixing part was designed to enhance mixing performance and was achieved using PDMS and MEMS technology. The designed pretreatment and mixing part showed high mixing efficiency even when the Type C micro-channel with a flow rate was 0.3 µL/min was used and analysis time of the proposed sensor took 4 min. The absorbance of blue-colorized saliva was measured through the LED and the photodiode to calculate the glucose concentration in the fluids. A 45° etched reflective surface was applied to the absorbance measurement part to increase the optical path length. The increase in the optical path length increased the sensitivity of the proposed LOC. The fabricated sensor was evaluated by measuring glucose concentration in the saliva and comparing the results with those obtained using an existing glucose sensor. According to previously reported research, patients with diabetes have a 170~180 mg/dL glucose concentration in blood and a 10~11 mg/dL glucose concentration in saliva [10,11]. Thus, a glucose sensor utilizing saliva must be able to detect glucose concentration of a few milligrams of fluid. We confirmed that the proposed sensor can measure a glucose concentration of a few milligrams after optimizations described above. The relationship between glucose concentration in the blood and that in the saliva was also confirmed using the proposed LOC. Performance characterization of the fabricated LOC showed that the proposed LOC is suitable as a portable device for continuous glucose monitoring.
